# 

**Published:** 1910-09

**Authors:** 


### CONTENTS.

ORIGINAL COMMUNICATIONS—

The Doctrine of Probabilities in the Early Diagnosis of Peritonitis. By R. M. Harbin, M. D., Rome, Ga. 273

Practical Aids in Medical and Surgical Diagnosis. By Lewis M. Gaines, A. B., M. D., Atlanta, Ga., 283

Chronic Non-Gonorrheal Infections of the Prostrate Gland. By Ddgar G. Ballenger 288

Hypertrophied Tonsils and Adenoids an Etiological Factor in Backward Children. By Geo. H. Cooper, M. D., Opelika, Ala., 293

Considerations in Surgical Convalescence. By H. P. Cole, M. D., Mobile, Ala., 298

A Note on the Nature of Neurasthenia. By Dr. Tom A. Williams, Washington, D. C., 303

American Association for Study and Prevention of Infant Mortality 305

EDITORIALS 307

NEWS AND NOTES 310

BOOK REVIEWS 319

READING NOTICES 322

